# Electron Delocalization Induced by 3d–4f Orbital Hybridization of Co─Ce Atom Pairs Toward Enhanced Electromagnetic Wave Attenuation and Antibacterial Performance

**DOI:** 10.1002/advs.74918

**Published:** 2026-03-18

**Authors:** Da Li, Yinyin Zhao, Yang Zhang, Cui Ni, Xiaoguang Zhao, Chongchang Zhou, Guoqiang Ren, Pengfei Liu, Xiubo Xie

**Affiliations:** ^1^ Ningbo Institute of Innovation for Combined Medicine and Engineering The Affiliated Lihuili Hospital of Ningbo University Ningbo China; ^2^ School of Environmental and Material Engineering Yantai University Yantai China; ^3^ Department of Electrical and Electronic Engineering The University of Hong Kong Hong Kong Hong Kong

**Keywords:** antibacterial performance, Co─Ce atomic pairs, electromagnetic wave absorption, electron structure modulation, orbital hybridization

## Abstract

Transition metal single atoms dispersed on nitrogen‐doped carbon (NC) can effectively manipulate local charge motions and reinforce polarization responses. However, the limited electron delocalization restricts the further improvements of electromagnetic wave absorption (EMA) capability for single atom systems. Herein, a novel Co─Ce dual atomic configuration anchored on the surface of NC matrix was constructed to regulate the local electron structure. The higher occupied states induced by the Co 3d‐Ce 4f orbital hybridization facilitate the formation of polarized electric fields, which in turn strengthens the polarization relaxation and partially repairs the effective electron conduction. Consequently, the CoCe@NC composites achieve a remarkable maximum absorption intensity of ‐85.1 dB and an expanded absorption bandwidth of 7.6 GHz at 1.7 mm. Meanwhile, the stronger inter‐orbital electron interactions promote the local charge transfer, endowing CoCe@NC systems with optimized POD‐like catalytic activity and enhanced antibacterial performance against *E. coli* and *S. aureus*. This work highlights the potential of 3d‐4f orbital hybridization engineering in designing advanced multifunctional EMA materials, which integrate EMW attenuation and antimicrobial properties to tackle multifaceted challenges.

## Graphical abstract

Co─Ce dual atomic configuration anchored on the surface of NC matrix has been constructed to regulate the local electron structure. The higher occupied states induced by the Co 3d‐Ce 4f orbital hybridization facilitate the formation of polarized electric fields, which in turn strengthens the polarization relaxation and partially repairs the effective electron conduction. Additionally, the stronger inter‐orbital electron interactions promote the local charge transfer, thereby optimizing POD‐like catalytic activity and enhancing antibacterial performance.

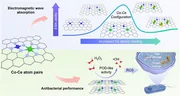

## 1 Introduction

The rapid upgrading of information technologies and communication equipment brings great convenience to social life, but also involves inevitable electromagnetic radiation and interference, which puts forward stringent design requirements for electromagnetic wave absorbing (EMA) materials [1, 2, 3, 4, 5]. Meanwhile, the resistance to microbial contamination for EMA materials is a growing concern in applications such as portable electronics and medical devices [6, 7]. Under such conditions, the development of multifunctional EMA systems integrating superior electromagnetic protection performance and antimicrobial capability has become an urgent demand [8, 9, 10].

Transition metal single‐atom (SA) materials with discrete energy level distribution and nearly 100% atomic utilizations offer a promising opportunity for the design of multifunctional EMA systems [11, 12, 13, 14]. In particular, the nitrogen doped carbon (NC) nanosheets loaded with SAs though M‐N‐C pathway have attracted extensive attention due to their robust metal‐support interactions and programmable local electron mobility [15, 16, 17, 18]. The large potential difference between metal SAs and the adjacent N/C atoms can trigger directional electron transfer, thus disrupting the inherent symmetry of electron cloud distribution within carbon lattice [19, 20, 21]. The resulting separation of positive and negative charge centers favors the formation of electric dipoles, which in turn optimizes the polarization relaxation behaviors under the excitation of incident electromagnetic waves (EMW). On the other hand, the M‐N‐C structure exhibits a similar configuration to the active sites of natural enzymes, thereby demonstrating enzyme‐like catalytic effects for generating reactive oxygen species (ROS) to kill the adherent bacteria [22, 23, 24, 25]. However, transition metal SAs sites typically exhibit D_4h_ symmetric coordination configurations, leading to insufficient electron delocalization, which significantly limits the EMA performance at C and X bands (4–12 GHz) [21]. Meanwhile, the symmetric configuration restricts the electron transfer capacity of active sites, hindering the enhancement of antibacterial efficiency for nanozyme materials.

Orbital hybridization refers to the effect of electron coupling occurring between different metal orbitals [26, 27, 28, 29]. By introducing atoms with distinct orbital characteristics to form dual atomic pairs with the host transition metal atoms (such as Fe, Co and Ni), it is expected to intensify the distortion degrees of local electron cloud. Rare earth (RE) elements with rich 4f orbitals and highly localized 4f electrons are promising candidates to construct dual atomic configurations, which can serve as effective electron reservoir to induce charge accumulation at transition metal atom sites [30, 31]. Among RE species, Ce element with high‐coordination structures is quite attractive owing to its unique electron configuration of [Xe]4f^1^5d^1^6s^2^, where the partially filled 4f orbital is occupied by only one electron [32, 33, 34, 35, 36]. This implies that when the 4f orbital of Ce atoms overlaps with the d orbital of transition atoms, the coupling of inter‐orbital electrons will be stronger, leading to enhanced electron transfer and optimized dielectric relaxation characteristics [37, 38, 39, 40]. However, it still remains a significant challenge to precisely control the coordination geometry between Ce and transition metal atoms at the atomic scale, which is crucial for optimizing electronic delocalization properties. Furthermore, the underlying mechanism linking between electron asymmetric distribution and dielectric properties/catalytic activity has yet to be fully understood.

Herein, we design a NC‐based EMA system with novel Co─Ce configurations through a facile adsorption‐pyrolysis approach, where the ∼10 nm Co nanodots and Co─Ce dual atomic sites are co‐dispersed on the surface of NC nanosheets. The integration of Ce atomic sites induces the orbital hybridization effects between Co 3d and Ce 4f orbitals, which promotes the local charge accumulation at Co sites via Ce─N─Co pathway. The intensified electron delocalization evokes enhanced polarization responses, thereby facilitating the EMW energy consumption. Meanwhile, the strong electronic coupling interactions between Co─Ce atom pairs and NC substrate promote local charge transfer, partially repairing the conductive loss ability. As a result, the well‐designed CoCe@NC absorber presents a maximum absorption intensity of ‐85.1 dB with an effective absorption bandwidth of 7.6 GHz at 1.7 mm. Moreover, the modulated electron structure effectively improves the POD‐like catalytic activities, endowing the CoCe@NC systems with excellent antibacterial efficiency. This work provides new insights into the synchronous regulation of electromagnetic protection and microbial resistance by exploiting 3d‐4f orbital hybridization engineering.

## 2 Results and Discussion

**FIGURE 1:**
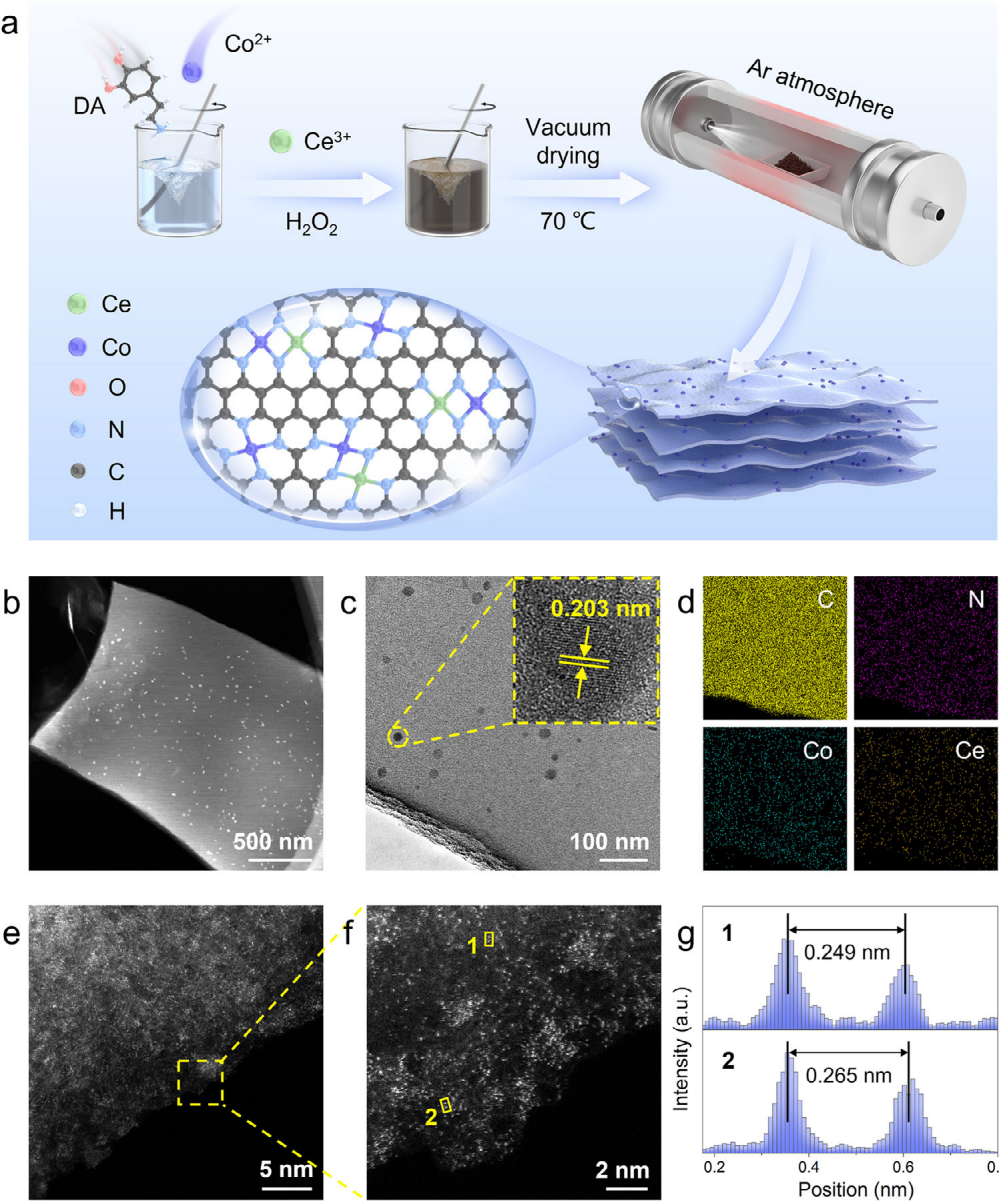
(a) Schematic illustration of the preparation procedure for CoCe@NC composites. (b) Dark field TEM image, (c) enlarged TEM image (the inset present the HRTEM image of the loaded nanodots), (d) elemental distribution mapping of the prepared CoCe@NC samples. (e,f) AC‐STEM images and (g) the corresponding intensity line profiles of dual atomic sites marked in zone 1 and zone 2 for CoCe@NC layers.

The CoCe dual‐atom composites were synthesized via a controllable adsorption‐pyrolysis strategy. As illustrated in Figure 1a, a certain amount of Ce^3+^ ions was added to the mixed solution containing dopamine (DA) and Co^2+^ ions, followed by the addition of hydrogen peroxide to induce the self‐polymerization of DA molecules. The dark brown mixture was then dried in a vacuum oven to yield the CoCe‐PDA precursor powder, which was subsequently converted into 2D nanosheets through a carbonization process under Ar atmosphere. The resulting CoCe composites with the addition of 0, 0.05, 0.07, 0.12, and 0.17 mmol Ce^3+^ ions were referred to as Co@NC, CoCe@NC‐1, CoCe@NC‐2, CoCe@NC‐3 and CoCe@NC‐4, respectively.

The morphology and microstructure of the yielding products have been investigated by transmission electron microscopy (TEM) and energy dispersive X‐ray spectroscopy (EDS) measurements. As observed in the dark field TEM image (Figure 1b), the CoCe@NC composites present an ultrathin flaky morphology, with well‐dispersed nanodots of ∼10 nm loaded on the surface. From the high‐resolution TEM image in Figure 1c, it can be found that the lattice fringe spacing of the nanodots is determined to be 0.203 nm, corresponding to the (111) crystal plane of fcc‐Co. This result matches well with the X‐ray diffraction (XRD) patterns (Figure S1), where three distinct diffraction peaks can be detected at 44.3°, 51.6°, and 75.9°, confirming that the nanodots are mainly Co species. Notably, several new weak peaks emerge at 25.3°, 30.9°, and 34.1° with the increase of Ce content, suggesting the formation of CeOx species in CoCe@NC composites. On the other hand, Co and Ce elements are identified to be uniformly distributed on the nanosheets (Figure 1d), indicating the successful loading of atomic Co and Ce sites. The spherical aberration corrected scanning transmission electron microscopy (AC‐STEM) have been further performed to verify the existence of Co and Ce dual single atoms (Figure 1e). Owing to the higher energy density than that of carbon species, Co and Ce atoms can be identified as isolated bright spots. Furthermore, Co─Ce atomic pairs can be clearly detected in the enlarged AC‐STEM image (Figure 1f), such as the dual atomic sites marked with yellow rectangles. The corresponding intensity line profiles demonstrate that the dual atom sites present different intensities (Figure 1g) over a distance of ∼0.25 nm, further confirming the formation of specific Co─Ce pairs. The above results demonstrate the co‐existence of Co nanodots and Co─Ce dual single atoms in the prepared CoCe@NC composites.

**FIGURE 2:**
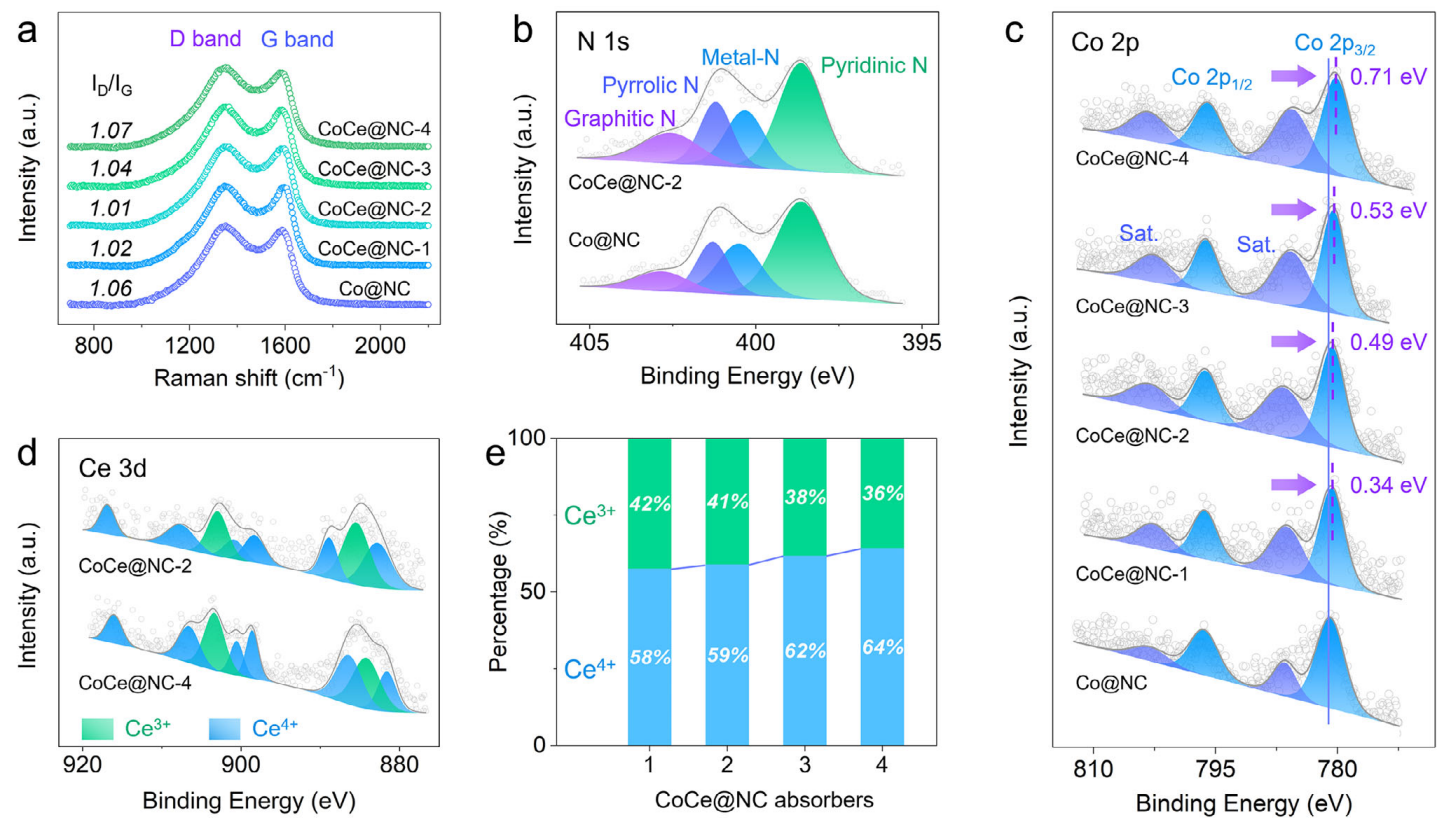
(a) Raman spectra, (b) N 1s, (c) Co 2p and (d) Ce 3d deconvoluted XPS spectra for CoCe@NC. (e) Ce^3+^ and Ce^4+^ contents in the prepared CoCe@NC composites.

In order to evaluate the graphitization degree of the carbon matrix, Raman spectra for the prepared CoCe@NC composites have been recorded in Figure 2a. Two typical peaks can be observed at about 1350 and 1595 cm‐^1^, which can be attributed to the D‐ and G‐band of carbon species [41]. With the growth of Ce element addition, the intensity ratios of D‐ and G‐band are firstly decreased and then increased. The smaller I_D_/I_G_ values for CoCe@NC‐1 and CoCe@NC‐2 suggest that the construction of Co─Ce atomic configurations can promote local charge redistribution, thereby facilitating electrons transfer between crystalline and amorphous graphite regions [7, 16]. Besides, CoCe@NC‐3 and CoCe@NC‐4 present higher I_D_/I_G_ values than that of CoCe@NC‐1 and CoCe@NC‐2, indicating that the graphitization degree of NC substrate gradually decreases with the emergence of CeO_x_ species. The generated CeO_x_ species can distort the arrangement of surrounding carbon atoms, hindering their ordered bonding during graphitization, thereby indirectly reducing the graphitization degree. The surface compositions of CoCe@NC systems are investigated on the basis of X‐ray photoelectron spectroscopy (XPS) measurements. As depicted in Figure 2b and Figure S2, the high‐resolution N 1s spectra can be fitted into four peaks including pyridinic N (398.4 eV), metal‐N (400.1 eV), pyrrolic N (401.1 eV) and graphitic N (402.4 eV). The existence of metal‐N bonds demonstrates that Co and Ce atoms are interacting with carbon substrate through Co─N─C and Ce─N─C pathways [22, 28, 42]. For the Co 2p spectra (Figure 2c), the deconvoluted peaks centered at 780.9 and 796.4 eV can be assigned to Co 2p_3/2_ and Co 2p_1/2_, respectively. After the introduction of Ce atoms, the orbital peaks of Co species exhibit negative shifts toward lower binding energy, indicating a directional electron flow from Ce to Co atoms. The XPS spectra for Ce elements are shown in Figure 2d and Figure S3, which can be deconvoluted into two sets of peaks involving Ce^3+^ and Ce^4+^ [6, 35]. The proportion of Ce^4+^ increase from 58% to 64%, indicating the formation of CeO_x_ species with increasing Ce loadings in CoCe@NC composites (1.01 wt.% for CoCe@NC‐1, 1.47 wt.% for CoCe@NC‐2, 2.39 wt.% for CoCe@NC‐3 and 3.16 wt.% for CoCe@NC‐4), which is consistent with the XRD results. The emerging CeO_x_ species can disrupt the carbon lattice continuity and reduce the number of mobile electrons [36, 38], thus diminishing the conductive loss ability. Electron paramagnetic resonance (EPR) spectra have been further employed to evaluate the electron interactions between Co and Ce atoms. As shown in Figure S4, the EPR spectra of Co@NC and CoCe@NC‐2 present a g value of 2.0024 and 2.0036, respectively. The g value of CoCe@NC‐2 is slightly higher than that of CoCe@NC, indicating that the introduction of Ce atoms modifies the electronic structure and coordination environment of Co atoms, thereby enhancing the interaction between electron spin and the applied external magnetic field. Besides, the characteristic peak intensity of CoCe@NC‐2 is significantly enhanced compared to Co@NC. This demonstrates that the Ce doping promotes electron transfer from Ce to Co sites, thereby increasing the number of unpaired electrons in Co 3d orbitals.

**FIGURE 3:**
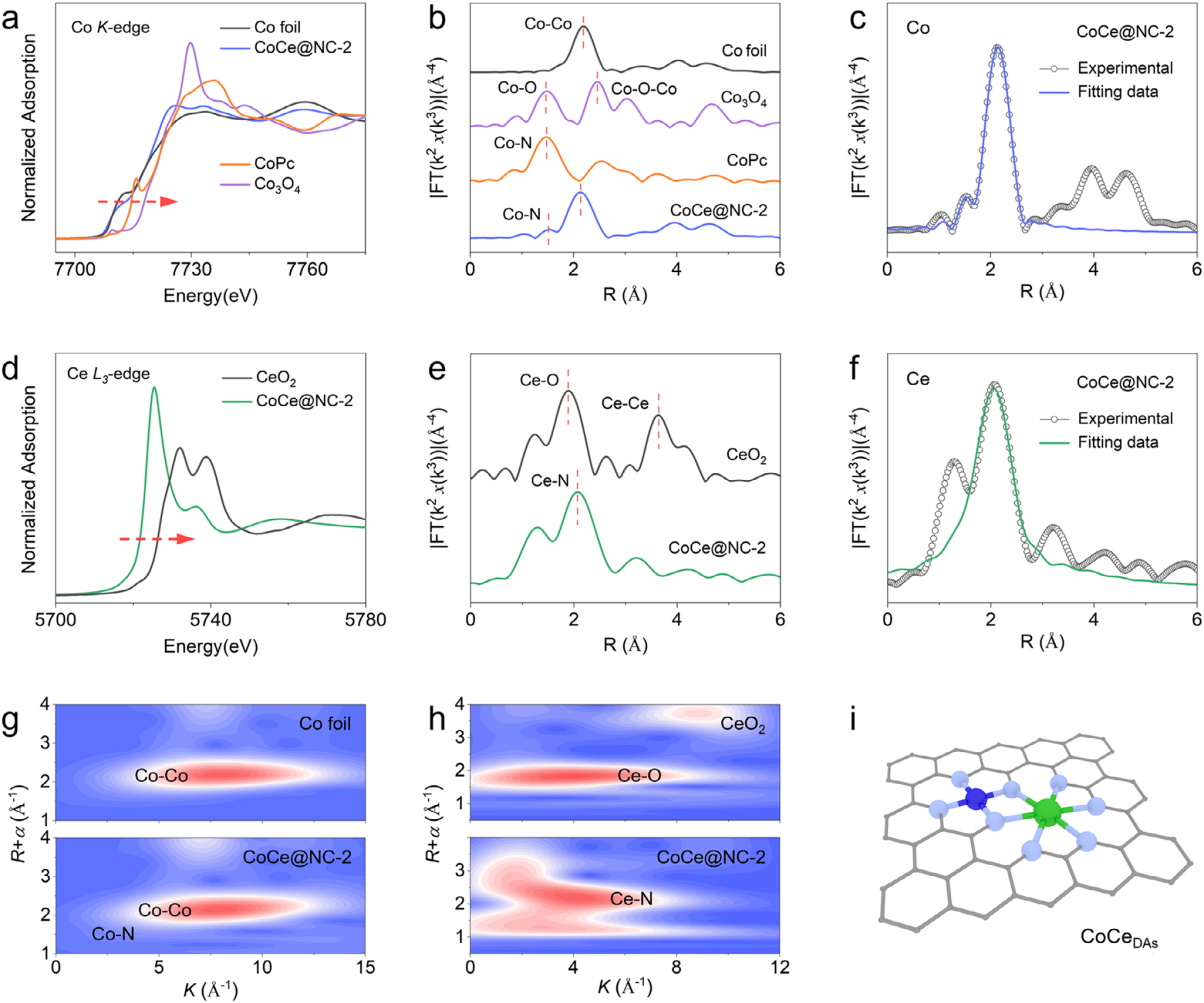
(a) Normalized Co K‐edge XANES spectra and (b) the corresponding Fourier transformations of EXAFS spectra at R space for CoCe@NC‐2 and cobalt standards (Co‐foil, Coc, and Co_3_O_4_). (d) Normalized Ce L_3_‐edge XANES spectra and e) the corresponding FT‐EXAFS spectra for CoCe@NC‐2 and CeO_2_. (c,f) Experimental and fitting EXAFS plots for Co and Ce elements in CoCe@NC‐2. (g) Co K‐edge and (h) Ce L_3_‐edge wavelet transform contour mapping for CoCe@NC‐2 and related standard samples. (i) Atomic structure of CoCe_DAs_ (Co─Ce dual atoms) configuration.

The X‐ray absorption near‐edge spectra (XANES) and extended X‐ray absorption fine structure (EXAFS) are conducted to investigate the valence states and coordination environments of Co and Ce species. The Co K‐edge XANES spectrum of CoCe@NC‐2 presents similar near‐edge absorption behaviors to that of Co foil (Figure 3a), suggesting that there is metallic Co particles loaded on NC layers. Meanwhile, the absorption edge position of CoCe@NC‐2 situates between Co foil and CoPc, indicating that the valence state of Co is between 0 and +2. The Fourier‐transformed EXAFS (FT‐EXAFS) spectra in Figure 3b reveal a predominant Co─Co scattering path at 2.11 Å in CoCe@NC composites, confirming the existence of metallic Co species, which matches well with the observation in TEM images. Besides, a characteristic Co─N scattering peak can be discerned at 1.53 Å, indicating the existence of dispersed Co atomic sites.

For Ce elements, the Ce L_3_‐edge adsorption threshold position of CoCe@NC‐2 presents a negative shift compared to that of CeO_2_ (Figure 3d), demonstrating a lower Ce valence state than +4 [37]. As shown in Figure 3e, a distinct Ce‐N scattering path can be observed at 2.02 Å, while the Ce‐Ce interaction between 3 and 4 Å is absent, implying the distribution of coordinatively isolated Ce sites [33, 34]. The well‐fitted EXAFS curves in R and K space (Figure 3c,f; Figure S5) further confirm the Co─N and Ce─N coordination in NC plane. In addition, as shown in the corresponding WT contour plots (Figure 3h,i; Figure S6), the scattering signal maxima of Co─N (5.1 Å) and Ce‐N (4.4 Å) paths for CoCe@NC‐2 exhibit distinct offsets compared to that of Co foil (7.2 Å) and CeO_2_ (3.9 Å), which manifest that there are significant interactions between Co and Ce atomic sites. Collectively, the above analysis validates the successful construction of Co─Ce configurations in CoCe@NC composites (Figure 3i).

**FIGURE 4:**
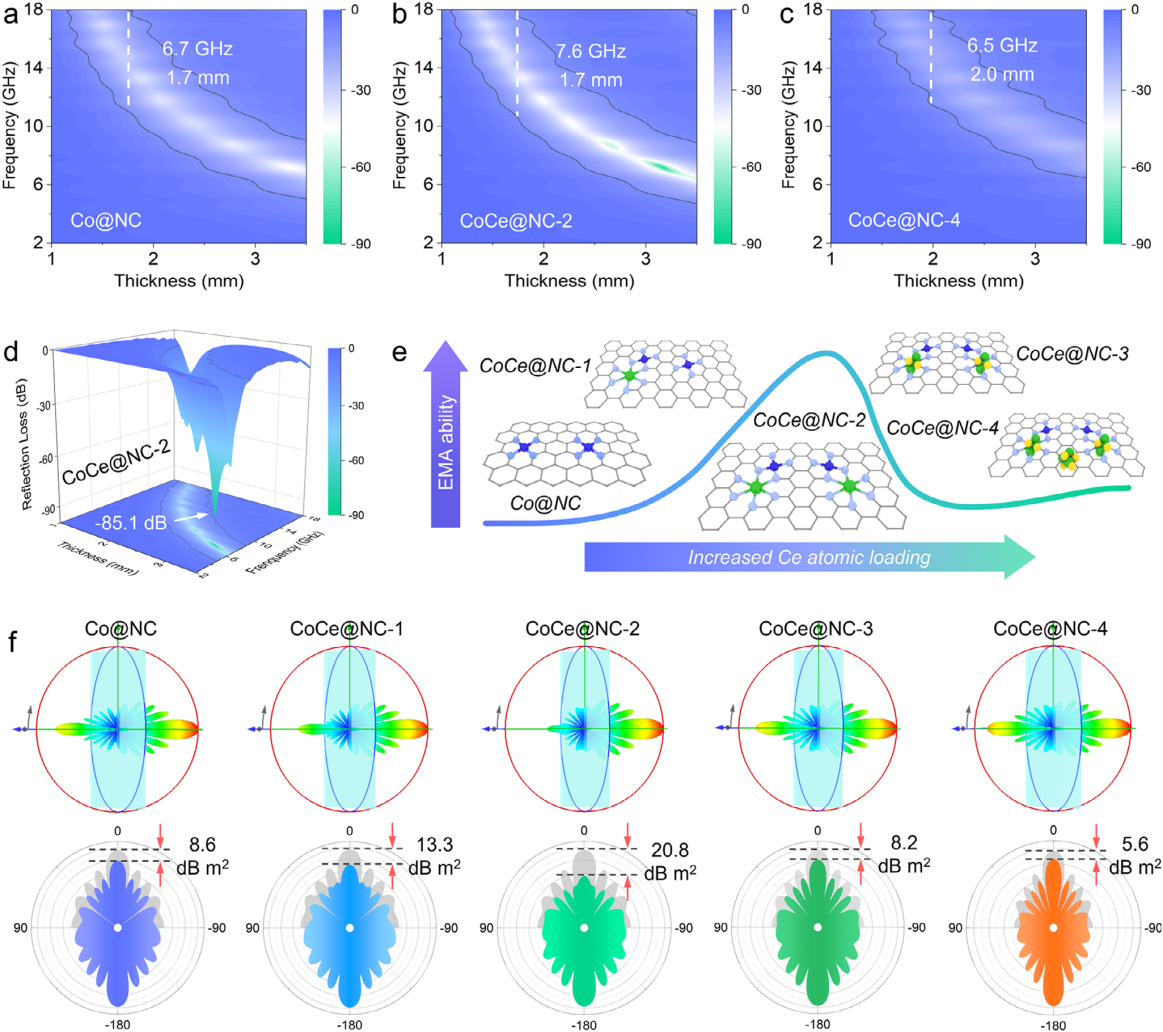
(a–c) 2D RL projections of Co@NC, CoCe@NC‐2 and CoCe@NC‐4, respectively. (d) 3D RL values of CoCe@NC‐2. (e) Evolution of EMA performance for CoCe@NC systems with increasing Ce loading. (sf) 3D RCS signal plots and the corresponding RCS signal values for CoCe@NC composites. The RCS signal in gray color derived from PEC.

The EMA performance of CoCe@NC composites has been evaluated based on reflection loss values (RL) and effective absorption bandwidth (EAB, corresponding to RL≤‐10 dB). Co@NC with Co single atom sites exhibits a minimum RL (RL_min_) value of ‐44.8 dB with an EAB of 6.7 GHz at 1.7 mm (Figure 4a; Figure S7). After the introduction of lower content of Ce element, the maximum absorption intensity of CoCe@NC‐1 increases to ‐48.9 dB, while the EAB expands to 7.0 GHz at 1.8 mm (Figure S8). With the increase of Ce loadings, the EAB can achieve 7.6 GHz at a matching thickness of 1.7 mm (Figure 4b). Meanwhile, the RL_min_ value of CoCe@NC‐2 further grows to ‐85.1 dB (Figure 4d). The greater absorption intensity and wider absorption bandwidth demonstrate that the construction of Co─Ce dual sites can significantly optimize the electromagnetic responses of CoCe@NC absorbers. However, the EMW attenuation abilities of CoCe@NC composites suffer a reduction when the Ce atom loading exceeds the saturated state. Specifically, CoCe@NC‐3 exhibits a RL_min_ value of ‐29.6 dB with an EAB of 6.6 GHz at 1.8 mm (Figure S8). For CoCe@NC‐4 with further increased Ce content, the EMA performance presents an evident degradation with a RL_min_ value of ‐24.2 dB and an EAB of 6.5 GHz at 2.0 mm (Figure 4c; Figure S7). The impairment of the absorption properties can be mainly attributed to the emerging CeO_x_ species, which trap the surrounding mobile electrons and confine the charge transfer paths. The evolution of EMA behaviors has been summarized in Figure 4e and Table S1, where CoCe@NC‐2 demonstrates optimal absorption intensity and bandwidth among CoCe@NC systems. Moreover, CoCe@NC‐2 composites also present distinct advantages both in RL_min_ and EAB values over other reported single‐atom absorbers (Figure S9 and Table S4), suggesting that Co─Ce configurations play a key role in regulating absorption properties.

Furthermore, the radar cross section (RCS) simulations under far‐field conditions have been carried out to assess the absorption efficiency of CoCe@NC absorbers for practical application scenarios, in which a perfect electrical conductor (PEC) is used as the substrate [1, 14, 15]. As shown in Figure 4f and Figure S10, the radar scattering signals from PEC substrate can be effectively suppressed after covering CoCe@NC coatings. In particular, CoCe@NC‐2 presents superior RCS signal attenuation performance with a maximum RCS reduction up to 20.8 dB m^2^ at 0°. These results highlight the promising potential of the Co─Ce configuration modulated absorber systems in future real‐world applications.

**FIGURE 5:**
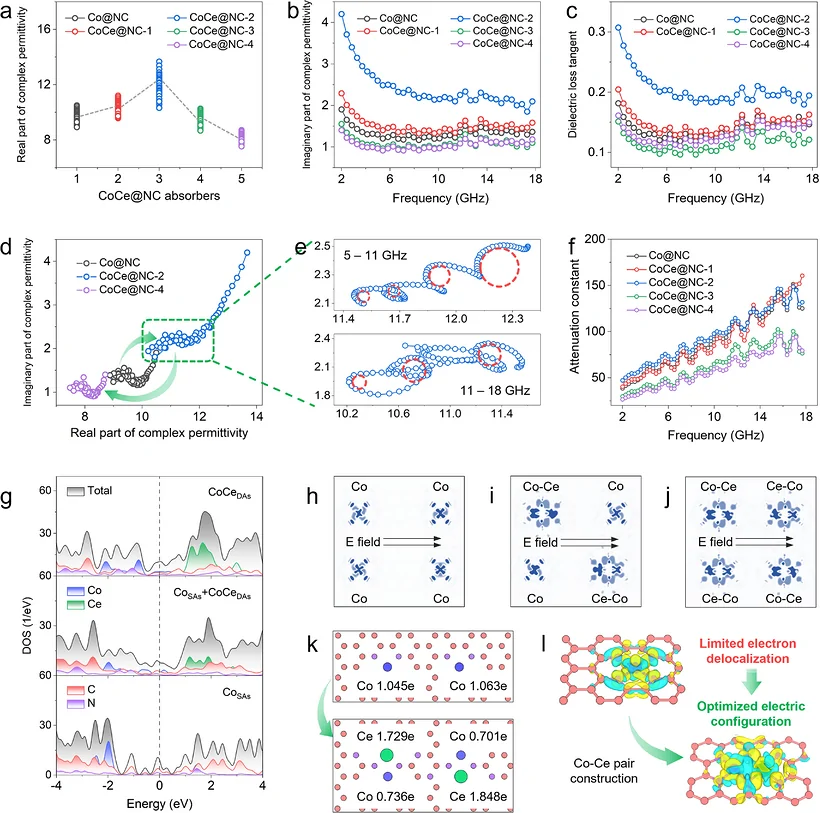
(a) Real part and (b) imaginary part of complex permittivity, (c) dielectric loss tangents, (d) Cole–Cole curves, (e) relaxation responses between 5–11 and 11–18 GHz, (f) attenuation constants for CoCe@NC composites. (g) Partial density of states (PDOS) for Co_SAs_, Co_SAs_+CoCe_DAs_, CoCe_DAs_ models. Differential charge density of (h) Co_SAs_, (i) Co_SAs_+CoCe_DAs_ and (j) CoCe_DAs_ models under electric field (the arrows represent the direction). The dark blue and light blue represent charge depletion and accumulation, respectively. (k) Calculated Bader charge values for Co sites before and after the introduction of Ce sites. (i) Schematic of electron delocalization in Co─Ce atomic configuration.

In order to elucidate the intrinsic EMW attenuation mechanisms, electromagnetic parameters involving the complex permeability (*µ_r_* = *µ′*−*jµ′′*) and complex permittivity (*ε_r_ = ε′−jε′′*) are investigated. Since the CoCe@NC systems exhibit close saturated magnetic moments and similar magnetic responses (Figures S11 and S12), we have concentrated on studying their dielectric characteristics. Generally, the real and imaginary part of the complex permittivity can be used to assess the energy storage and charge attenuation abilities, respectively [43]. As depicted in Figure 5a, the *ε′* values increase from 10.49–8.92 to 11.21–9.58 after doping Ce atoms in NC matrix. With the increase of Ce addition, the *ε′* values further raise to 13.67–10.32. The growing trend can be attributed to the formation of Co─Ce atomic configurations dispersed in NC layers. Benefitting from the hybridization effect of Co 3d and Ce 4f orbitals, the local electron structure can be effectively modulated, leading to improved charge densities and enhanced electronic interactions between the Co species and NC matrix. As the mass fraction of Ce element continues to increase beyond the saturated concentration for the formation of Ce atomic sites, the CeO_x_ species begin to appear, resulting in a decrease in *ε′* values. Consequently, the *ε′* values drop to 10.27–8.73 for CoCe@NC‐3 and 8.71–7.60 for CoCe@NC‐4 composite, and similar evolution trend can be observed for the *ε′′* values of CoCe@NC systems (Figure 5b). It can be inferred that the CeO_x_ species reduce the number of mobile electrons, thereby compromising the dielectric loss capability of CoCe@NC systems, leading to diminished EMW attenuation.

The dielectric response capability can be evaluated according to the dielectric loss tangents (tan *δ_ε_*), which are illustrated in Figure 5c. It can be found that CoCe@NC‐2 with high density Co─Ce configurations exhibit largest tan *δ_ε_* values among the whole frequency range, suggesting that the 3d‐4f orbital hybridization can contribute significantly to the EMW energy consumption. Meanwhile, the improved dielectric properties also facilitate the coupling with magnetic features, leading to optimized interfacial impedance matching. The |Z_in_/Z_0_| values are usually utilized to evaluate the impedance matching degrees, where Z_in_ and Z_0_ represent the impedance for absorber materials and free space, respectively. When the |Z_in_/Z_0_| values are close to 1, the incident EMW can propagate into the interior of absorbers instead of being reflected at the air‐absorber interface [2, 15]. As shown in the 2D |Z_in_/Z_0_| contour plots (Figure S13), CoCe@NC‐2 presents larger matching area than other CoCe@NC absorbers, which is as high as 17.9% of the domains. A better impedance matching facilitates CoCe@NC‐2 to capture more of the incident EMW and dissipate their energy by means of magnetic resonance, conductive loss, polarization relaxation and so on.

Furthermore, the polarization characteristics of CoCe@NC composites are investigated on the basis of Cole–Cole semicircle curves. With the construction of Co─Ce dual sites, CoCe@NC‐2 presents a longer and steeper near‐linear tail than that of Co@NC (Figure 5d), indicating a more pronounced conductive loss behavior. Besides, multiple distinct semicircles representing sufficient relaxation processes can be observed within the frequency range of 5–18 GHz (Figure 5e). This phenomenon demonstrates that the polarization losses play a significant role in EMW dissipation over C, X and Ku bands [8, 35]. Notably, both the length of the near‐linear tail and the radius of the semicircles of the Cole–Cole plots for CoCe@NC‐4 decrease sharply, suggesting that the emerging CeO_x_ species are not conducive to the improvement of EMW absorption, which is further verified by the attenuation constants (*α*) shown in Figure 5e. Based on the above analysis, it can be reasonably inferred that the enhanced dielectric loss ability and improved impedance matching derived from the 3d‐4f orbital hybridization of Co─Ce atom pairs are the primary accounts for the optimized EMW attenuation performance.

Density functional theory (DFT) simulations are performed to unveil the modulation mechanisms of 3d‐4f orbital hybridization on dielectric responses in CoCe@NC composites. Based on the XPS and XANES results, three atomic models including Co_SAs_, Co_SAs_+CoCe_DAs_ and CoCe_DAs_ have been developed. As depicted in Figure 5g, there are pronounced overlap regions for the partial density of state (PDOS) of Co and Ce, indicating the strong electronic coupling between Co 3d and Ce 4f orbitals. Besides, the PDOS of Ce 4f grows with the increase of Ce loading, which in turn contributes to the improvement of total density of state near Fermi level. The higher occupied states induced by the strong inter‐orbital hybridization contribute to driving local charge rearrangement and promoting electron migration, which enhances the polarization responses and improves macroscopic conductive properties [1, 44]. The electron motions surrounding Co and Ce sites are further investigated by the calculated differential charge density (Figure 5h–j; Figure S14). Under the applied electric filed with a certain direction, the local electrons tend to flow from Ce to Co atoms in both the Co─Ce and Ce─Co configurations. This suggests that the introduction of Ce atomic sites exacerbates intrinsic charge density distortion and accelerates the interfacial electron delocalization [32]. The separation of positive and negative charge centers causes the formation of electric dipoles, inducing enhanced polarization responses, which are responsible for the improved EMA capability [15]. Moreover, the corresponding Bader charges are calculated to explicitly illustrate the electron redistribution in Co─Ce configurations (Figure 5k). Owing to the high energy level of 4f orbitals, Ce atoms can act as electron pumps to promote the rapid directional electron transfer. As a result, the Bader charges are +0.736 and +0.701 for Co sites and +1.729 and +1.848 for Ce sites. Notably, compared to the isolated Co single atoms, the construction of Co─Ce dual sties results in net change values of ‐0.309 and ‐0.362, further confirming the charge accumulation at Co sites by means of Ce─N─Co bridge [33]. Meanwhile, the asymmetric local charge distributions are able to induce stronger electronic coupling interactions between Co─Ce sites and NC matrix, which promotes carrier motions along the carbon lattice and partially repairs the effective electron conduction [45]. The above data verify that the 3d‐4f orbital hybridization of Co─Ce atomic configurations can induce pronounced electron delocalization (Figure 5l), thus optimizing the dielectric loss characteristics and ultimately boosting EMW energy consumption.

**FIGURE 6:**
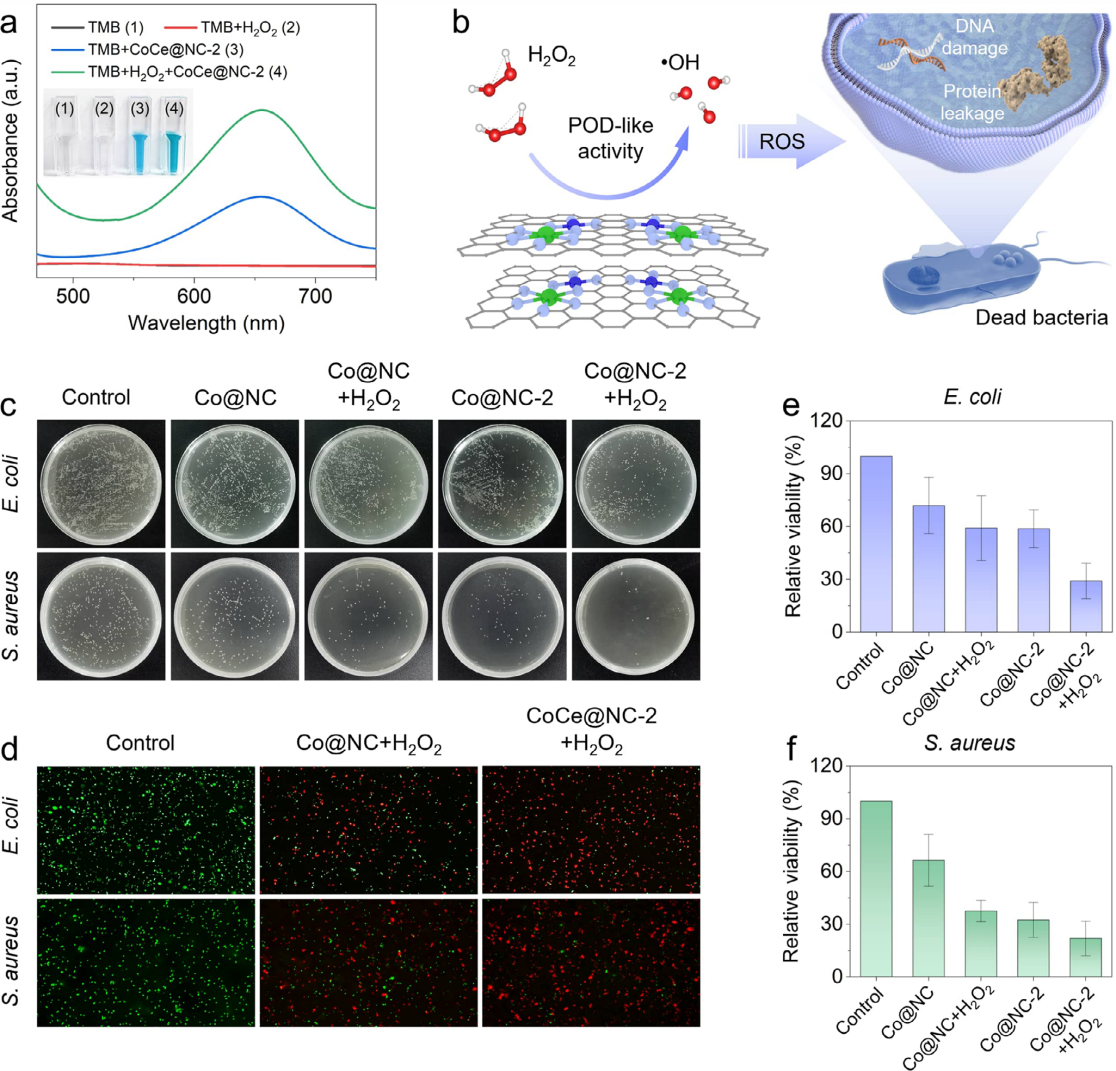
(a) POD‐like catalytic behaviors of CoCe@NC‐2 under different conditions using TMB probe (1 mm). (b) Schematic illustration of antibacterial mechanism for CoCe@NC composites. (c) Digital photos of *E. coli* and *S. aureus* colonies after different treatments. (d) The corresponding confocal images of live/dead stained *E. coli* and *S. aureus* colonies. The green and red color represent the live and dead bacterial, respectively. Relative viabilities of (e) *E. Coli* and (f) *S. aureus* colonies.

To explore the application potential of CoCe@NC composites to resist microbial corrosion under challenging conditions, the corresponding in vitro antibacterial performance have been investigated. The peroxidase‐like (POD‐like) catalytic performance are firstly evaluated by UV–vis measurements using 3,3′,5,5′‐tetramethylbenzidine (TMB) probe. The TMB molecule can be oxidized to oxTMB with blue color under the existence of ·OH radicals, thereby allowing a qualitative determination of the catalytic activity [24, 46, 47]. As depicted in Figure 6a, a clear absorption peak can be observed in the TMB+CoCe@NC‐2 group and a blue color can be detected in the corresponding cuvette, whereas it is absent in TMB and TMB+H_2_O_2_ groups, suggesting the generation of ·OH upon the addition of CoCe@NC‐2 composites. Moreover, a more pronounced absorption peak and a deeper bule color can be discerned for the TMB+H_2_O_2_+CoCe@NC‐2 group, which indicates the favorable POD‐like catalytic efficiency of CoCe@NC‐2. Specifically, Co─Ce atom pairs promote electron delocalization through 3d–4f orbital hybridization, thereby optimizing electron distribution at catalytic active sites. This process enhances the interaction between Co sites and H_2_O_2_, reduces reaction activation energy, and consequently boosts the POD‐like activity. Therefore, it can be inferred that the orbital hybridization between Co and Ce sites overcomes the “activity‐selectivity” limitations inherent in single‐atom materials. This electronic interaction optimizes both the efficiency and selectivity of ROS generation. The generated ROS can interfere or impede bacterial metabolic processes, causing DNA damage and protein leakage, thus creating effective sterilization of bacteria (Figure 6b) [23, 25].

The plate colony counting assays are further used to verify the antibacterial performance of the prepared CoCe@NC systems. For the in vitro antibacterial experiments, the Gram‐negative Escherichia coli (*E. coli*) and Gram‐positive Staphylococcus aureus (*S. aureus*) colonies have been selected as the microorganism models. The pH value and H_2_O_2_ concentration in the incubation environment are set to 4.0 and 1 mm, respectively. The bacterial strains have been organized into five different groups, including the control, Co@NC, Co@NC+H_2_O_2_, CoCe@NC‐2, and CoCe@NC‐2+H_2_O_2_ groups. As shown in Figure 6c, a similar degree of colony reduction can be observed in Co@NC+H_2_O_2_ and CoCe@NC‐2 groups, which is superior to the inhibition effect of the Co@NC group. Meanwhile, a more pronounced colony reduction in CoCe@NC‐2+H_2_O_2_ group can be observed, demonstrating the construction of Co─Ce configurations is conductive to the improvement of the antibacterial properties. For the CoCe@NC‐2+H_2_O_2_ group, the relative viability of *E. coli* and *S. aureus* are only 29.0% and 21.9%, respectively (Figure 6e,f). These results can be further verified by the live/dead bacterial staining tests (Figure 6d), where the green and red color represent the live and dead bacterial, respectively. In the CoCe@NC‐2+H_2_O_2_ group, almost all the *E. coli* and *S. aureus* bacteria have been marked in red after the incubation, confirming the generated ROS severely damage the cell structures and result in the inactivation of bacteria. The above results demonstrate that the modulation of the local electronic structure through the hybridization of Co 3d and Ce 4f orbitals can effectively optimize the enzymatic catalytic activity, leading to the superior antibacterial performance for CoCe@NC composites.

## 3 Conclusion

In summary, the Co─Ce dual atomic pairs have been successfully constructed on the surface of NC matrix through a feasible adsorption‐annealing strategy. The corresponding DFT simulations confirm that the 3d–4f orbital hybridization between Co and Ce sites intensifies the local electron cloud distortion and promotes spatial charge rearrangement. The regulated electron delocalization optimizes the dielectric polarization characteristics, which in turn exacerbate the EMW energy consumption. Meanwhile, the strong electronic interactions between Co─Ce configurations and NC substrate facilitate charge transfer along carbon lattice, thereby enhancing conductive losses. Consequently, the well‐designed CoCe@NC absorber presents a minimum RL value of ‐85.1 dB with favorable EAB of 7.6 GHz at 1.7 mm. Moreover, the modulated local electron environments optimize the POD‐like catalytic activity for CoCe@NC composites, which promotes the generation of ROS species and boosts the antibacterial potential against *E. coli* and *S. aureus*. This study paves a way for designing multifunctional EMA materials integrating EMW attenuation and microbial resistance through 3d‐4f orbital hybridization engineering.

## Author Contributions

Da Li: Investigation (Lead); Writing‐ review&editing (Equal). Yinyin Zhao, Yang Zhang, Cui Ni, Xiaoguang Zhao, Chongchang Zhou: Investigation (Supporting). Guoqiang Ren, Pengfei Liu: Writing‐ review& editing (Equal). Xiubo Xie: Conceptualization (Lead); Writing‐ review&editing (Equal). The precursor synthesis, DFT simulations and data analysis were performed at the Affiliated Lihuili Hospital of Ningbo University. The precursor carbonization, VNA and TEM measurements were conducted at Yantai University.

## Funding

We acknowledge the funding from Ningbo Yongjiang Talent Program under Grant No. 2024A‐421‐G, Ningbo Major Research and Development Plan Project (Grant No. 2025Z170), Ningbo Public Welfare Science and Technology Project under Grant No. 2025S185, Natural Science Foundation of Shandong Province under Grant No. ZR2025MS945, the Excellent Youth Innovation Team Project for Higher Education Institutions of Shandong Province of “Innovative Team for Key Materials in Hydrogen Energy and Electromagnetic Wave Absorption” and National Natural Science Foundation of China under Grant No. 52101274, Key Research and Development Projects of Shandong Province under Grant No. 2024TSGC0402.

## Conflicts of Interest

The authors declare no conflicts of interest.

## Supporting information




**Supporting File**: advs74918‐sup‐0001‐SuppMat.docx

## Data Availability

The data that support the findings of this study are available from the corresponding author upon reasonable request.
