# Supplementary material for: Electron Delocalization Induced by 3d–4f Orbital Hybridization of Co─Ce Atom Pairs Toward Enhanced Electromagnetic Wave Attenuation and Antibacterial Performance
Source: Adv Sci (Weinh). 2026 Mar 18;13(31):e74918. doi: 10.1002/advs.74918 (PMC13588078; doi:10.1002/advs.74918)
Supplement: Supplementary file 1 [file ADVS-13-e74918-s001.docx]

Supporting Information

**Title:** Electron delocalization induced by 3d-4f orbital hybridization of Co-Ce atom pairs towards enhanced electromagnetic wave attenuation and antibacterial performance

Da Li^a^, Yinyin Zhao^a^, Yang Zhang^a^, Cui Ni^b^, Xiaoguang Zhao^c^, Chongchang Zhou^a^, Guoqiang Ren^a^*, Pengfei Liu^a^*, Xiubo Xie^b^*

Da Li, Yinyin Zhao, Yang Zhang, Chongchang Zhou, Guoqiang Ren, Pengfei Liu

Ningbo Institute of Innovation for Combined Medicine and Engineering, The Affiliated Lihuili Hospital of Ningbo University, Ningbo, 315040, China

Email: lhlrenguoqiang@nbu.edu.cn; lhlliupengfei@nbu.edu.cn

Cui Ni, Xiubo Xie

School of Environmental and Material Engineering, Yantai University, No. 30 Qingquan Road, Yantai, 264005, China

Email: xiuboxie@ytu.edu.cn

Xiaoguang Zhao

Department of Electrical and Electronic Engineering, The University of Hong Kong, Hong Kong, SAR, 999077, China

**Experimental section**

*Materials*: Dopamine hydrochloride (DA, 98%), Cobalt nitrate hexahydrate (Co(NO_3_)_2_•6H_2_O, 99%), and Cerium(III) nitrate hexahydrate (CeN_3_O_9_·6H_2_O) were purchased from Maclin. Hydrogen peroxide solution (H_2_O_2_, 30 wt%) were purchased from Shanghai Maokang Biotechnology Co., Ltd.. All the chemicals were used directly without further purification.

*Preparation of CoCe@NC composites*: 1.0 g of dopamine was dissolved in 20 mL of deionized water and stirred for 30 min, followed by the addition of 0.6 g of Co(NO_3_)_2_•6H_2_O and stirring for another 30 min. A certain amount of Ce(NO_3_)_3_•6H_2_O and 1 mL of H_2_O_2_ was then added to the mixed solution, and stirring was continued until the solution turned deep brown. The mixture was subsequently transferred to a vacuum oven and dried overnight at 60 ℃. The resulting solid was ground into powder and placed in a tube furnace for pyrolysis under Ar atmosphere with a heat rate of 5 ℃/min. After holding at 800°C for 2 h and cooling to room temperature, the CoCe@NC composites were obtained. The products with Ce(NO_3_)_3_•6H_2_O addition amount of 0, 20, 30, 50 and 70 mg were labelled as Co@NC, CoCe@NC-1, CoCe@NC-2, CoCe@NC-3 and CoCe@NC-4, respectively.

*Characterizations*: The composition analysis were conducted by X-ray diffraction spectra (XRD, Rigaku SmartLab SE) with Cu Kα radiation source, and Raman spectroscopy (Thermo Scientific DXR). The interfacial chemical information was recorded by X-ray photoelectron spectroscopic (XPS, Krayos AXIS Ultra DLD) measurements with an Al Kα X-ray source. The coordination information was revealed by X-ray absorption spectroscopy (XAS) with 02B02 beamline. Inductively coupled plasma optical emission spectrometry (ICP-OES, Agilent 720ES) was performed to determine the Ce content. The morphology and microstructures of CoCe@NC composites were measured based on transmission electron microscopy (TEM, JEM-F200). The dispersed single atoms on carbon matrix were characterized by high angle annular dark-field scanning transmission electron microscopy (HAADF-STEM, JEOL ARM200F). The saturation magnetization (*M_s_*) and coercivity (*H_c_*) were measured by vibrating sample magnetometer (VSM, LakeShore 7404).

*Electromagnetic Measurement*: The EM properties for CoCe@NC absorbers were recorded by vector network analyzer (VNA, Agilent E5071c), while the loading contents were 40 wt%. In a typical test process, the samples were compressed into a toroidal ring with an outer diameter of 7.0 mm, an inner diameter of 3.0 mm, and a thickness of 2.0 mm. The reflection loss values of the absorbers were calculated on the basis of the transmission line theory through the following equations:^[1,2]^

$Z_{in}=Z_{0}\sqrt{\frac{\mu_{r}}{\varepsilon_{r}}}\tan h\left[ j\left( \frac{2\pi fd}{c} \right)\sqrt{\varepsilon_{r}\mu_{r}} \right]$	(1)

$RL=20\log_{10} \frac{\left| Z_{in}-Z_{0} \right|}{\left| Z_{in}+Z_{0} \right|}$	(2)

where *Z*_in_ and *Z*_0_ represent the input impedance and free space impedance, *ε_r_* and *μ*_r_ represent the complex permittivity and permeability, *f*, *d* and *c* demonstrate the EMW frequency, absorber thickness, and speed of light, respectively.

The attenuation constant (*α*), which is usually used to evaluate the EMW dissipation capability of absorber systems, can be calculated based on the following equation:^[3]^

$\alpha= \frac{\sqrt{2}\pi f}{c}\sqrt{\left( \mu^{''}\varepsilon^{''}-\mu^{'}\varepsilon^{'} \right)+\sqrt{\left( \mu^{''}\varepsilon^{''}-\mu^{'}\varepsilon^{'} \right)^{2}+\left( \mu^{'}\varepsilon^{''}+\mu^{''}\varepsilon^{'} \right)^{2}}}$	(3)

where ε′ and ε′′ stand for the real and imaginary part of the complex permittivity, μ′ and μ′′ are the real and imaginary part of the complex permeability, *f* and *c* present the frequency and vacuum velocity of light, respectively.

*RCS simulation*: In the radar cross section (RCS) simulation, a 180 × 180 × 1 mm^3^ perfect electric conductor (PEC) plate was set as the calculation model substrate. The obtained CoCe@NC absorbers were coated on the top of PEC matrix. Under far-field excitation, the RCS scattering signals can be evaluated based on the following equation:^[3]^

$\sigma\left( dB m^{2} \right)=10log((4\pi S/\lambda^{2})\mid E_{s}/E_{i}\mid)$	(4)

Where *S* and *λ* demonstrate the simulation model area and the EMW wavelength, *Es* and *Ei* represent the electric field intensity of the scattered wave and incident wave, respectively.

*DFT simulation*: Vienna *ab initio* simulation package (VASP) based on the plane-wave basis sets were utilized to perform the Density functional theory (DFT) calculations.^[4,5]^ The exchange-correlation potential was treated by using a generalized gradient approximation (GGA) with the Perdew-Burke-Ernzerhof (PBE) parametrization.^[6]^ The spin-polarized DFT+U correction with a typical *U* =3.32 eV value was applied to strongly correlated Co atoms.^[7,8]^ The calculated models were fully relaxed until the maximum force on each atom was less than 0.02 eV/Å, and the energy convergent standard was 10^-5^ eV. During the structural optimization process, an electric field with the intensity of 0.1 eV/Å was applied.

*In Vitro Antibacterial evaluation*: The antibacterial performance of CoCe@NC composites were evaluated by plate counting method using Escherichia coli (*E. coli*) and Staphylococcus aureus (*S. aureus*) colonies. The bacteria incubation were conducted in liquid medium consisting of 1% tryptone, 0.5% yeast extract and 1% NaCl, which were kept shaking under 120 rpm ⋅ min^-1^ for 18 h at 37°C. The bacteria were collected by centrifugation and diluted with sterile PBS solution to 10^6^ CFU mL^-1^ (CFU represents the colony-forming unit). Then, 500 μg CoCe@NC samples were mix with 1 mL of the bacterial solution and incubated in a thermostatic oscillator at 37 °C for 4 h. 1 mM of H_2_O_2_ was added to the corresponding groups when the bacterial strains were incubated for 2 h. Finally, 0.5mL of diluted bacterial solution was uniformly spread on Luria-Bertani (LB) solid medium and incubated at 37°C for 12 h, which were then used for the relative viability counting.

**Figure S1.** XRD patterns of the prepared CoCe@NC composites.

**Figure S2.** High-resolution N 1s XPS spectra of CoCe@NC-1, CoCe@NC-3 and CoCe@NC-4.

**Figure S3.** High-resolution Ce 3d XPS spectra of CoCe@NC-1 and CoCe@NC-3.

**Figure S4.** EPR spectra for Co@NC and CoCe@NC-2 composites.

**Figure S5.** The fitting EXAFS curves of a) Co and b) Ce in CoCe@NC-2 at K space.

**Figure S6.** The WT plots for a) CoPc and b) Co_3_O_4_.

**Figure S7.** The 3D RL plots for a) Co@NC and b) CoCe@NC-4.

**Figure S8.** The EMA performance for a, b) CoCe@NC-1 and c, d) CoCe@NC-3.

**Figure S9.** Comparison of the EMA performance of CoCe@NC-2 with previously reported absorber systems.

**Figure S10.** The 3D RCS scattering signal of PEC substrate.

**Figure S11.** The magnetic properties for the prepared CoCe@NC composites.

**Figure S12.** a) The complex permeability and b) magnetic loss tangents for the prepared CoCe@NC composites.

**Figure S13.** The 2D impedance matching plots for CoCe@NC composites, where the dashed and solid line represent the 0.8 and 1.2 contour lines, respectively.

**Figure S14.** The calculated differential charge density for a) Co_SAs_, b) Co_SAs_ + CoCe_DAs_ and c) CoCe_DAs_ models.

**Table S1.** Summary on the material properties of CoCe@NC samples.

| **CoCe@NC**  **absorbers** | **RL_min_**  **(dB)** | **EAB**  **(GHz)** | **Matching**  **Thickness**  **(mm)** | ***M_s_***  **(emu/g)** | ***H_c_***  **(Oe)** | **Ce**  **Content**  **(wt%)** | **Ce added amounts**  **(mg)** | **Filler ratio**  **(wt%)** |
| --- | --- | --- | --- | --- | --- | --- | --- | --- |
| **Co@NC** | -44.8 | 6.7 | 1.7 | 25.15 | 0.51 | - | - | 40 |
| **CoCe@NC-1** | -48.9 | 7.0 | 1.8 | 23.17 | 0.49 | 1.01 | 20 | 40 |
| **CoCe@NC-2** | -85.1 | 7.6 | 1.7 | 23.64 | 0.52 | 1.47 | 30 | 40 |
| **CoCe@NC-3** | -29.6 | 6.6 | 1.8 | 23.25 | 0.55 | 2.39 | 50 | 40 |
| **CoCe@NC-4** | -24.2 | 6.5 | 2.0 | 22.34 | 0.66 | 3.16 | 70 | 40 |

**Table S2.** EXAFS fitting parameters at the Co K-edge for various samples（*Ѕ*_0_^2^=0.76）

| **Samples** | **Shell** | **CN** | **R(Å)** | **σ^2^** | **ΔE_0_** | **R factor** |
| --- | --- | --- | --- | --- | --- | --- |
| **Co foil** | Co-Co | 12* | 2.49±0.01 | 0.0063 | 7.6±0.3 | 0.0017 |
| **CoCe@NC-2** | Co-N | 1.3±0.3 | 1.97±0.02 | 0.0080 | -1.1±0.7 | 0.0035 |
|  | Co-Co | 6.7±0.2 | 2.49±0.01 | 0.0059 |  |  |

*^a^CN*: coordination numbers; *^b^R*: bond distance; *^c^σ*^2^: Debye-Waller factors; *^d^*Δ*E*_0_: the inner potential correction. *^e^R* factor: goodness of fit.

**Table S3.** EXAFS fitting parameters at the Ce L_3_-edge for various samples（*Ѕ*_0_^2^=0.82）

| **Samples** | **Shell** | **CN** | **R(Å)** | **σ^2^** | **ΔE_0_** | **R factor** |
| --- | --- | --- | --- | --- | --- | --- |
| **CeO_2_** | Ce-O | 7.0±0.4 | 2.33±0.01 | 0.0049 | 6.7±0.8 | 0.0140 |
|  | Ce-Ce | 5.7±0.5 | 3.84±0.01 | 0.0001 |  |  |
| **CoCe@NC-2** | Ce-N/O | 6.2±0.4 | 2.57±0.02 | 0.0199 | 7.2±1.0 | 0.0186 |

*^a^N*: coordination numbers; *^b^R*: bond distance; *^c^σ*^2^: Debye-Waller factors; *^d^*Δ*E*_0_: the inner potential correction. *R* factor: goodness of fit.

**Table S4.** Summary on the EMA performance of previous reported absorber systems and CoCe@NC-2 in this work.

| **Absorbers** | **RL_min_ (dB)** | **EAB (GHz)** | **Thickness (mm)** | **References** |
| --- | --- | --- | --- | --- |
| Fe-N-rGO | -74.1 | 7.1 | 1.9 | *Nano-Micro Lett.* 2025, 17, 46 |
| La-N-Cl | -53.3 | 6.2 | 1.9 | *Adv. Funct. Mater.* 2024, 34, 2313483 |
| Co-N_4_-O/NCF | -45.8 | 4.8 | 1.6 | *Nano-Micro Lett.* 2024, 16, 240 |
| Ni-NC/MXene | -52.7 | 6.4 | 2.2 | *Nat. Commun.* 2024, 15, 9077 |
| Ni-SAs/HPCF-3/BP | -43.8 | 6.8 | 3.0 | *Adv. Funct. Mater.* 2026, 36, e19604. |
| Fe-SNC-T | -40.6 | 4.9 | 2.0 | *Adv. Funct. Mater.* 2025, 35, 2413784. |
| Co1+Cs/NGC-2 | -54.3 | 7.0 | 2.0 | *Adv. Funct. Mater.* 2024, 33, 2304442. |
| CeN_4_O_4_S_1.5_ | -51.0 | 4.4 | 1.5 | *Adv. Funct. Mater.* 2025, 35, 2504372 |
| LEMO@CNF | -46.7 | 3.5 | 5.2 | *Sci. Adv.* 2025, 11, eadz2218 |
| HCNF-Er | -57.8 | 3.0 | 3.7 | *Adv. Funct. Mater.* 2025, 35, 2502952. |
| SiOC-Fe-CN | -58.0 | 5.9 | 1.6 | *Adv. Mater.* 2026, e21533 |
| 2D-Co@NC-D | -26.7 | 5.6 | 1.8 | *Nat. Commun.* 2025,16, 202 |
| CoCe@NC-2 | -85.1 | 7.6 | 1.7 | **This work** |

**References**

[1]	J. Wen, S. Hui, Q. Chang, G. Chen, L. Zhang, X. Fan, K. Tao, H. Wu, *Adv. Funct. Mater.* **2024**, *34*, 2410447.

[2]	Z. Gao, A. Iqbal, T. Hassan, S. Hui, H. Wu, C. M. Koo, *Adv. Mater.* **2024**, *36*, 2311411.

[3]	N. Wang, X. Kou, L. Zhong, G. Zeng, A. Farid, X. Zhou, Q. Wang, D. Xi, G. Su, H. Huang, Y. Zhao, *Sci. Adv.* **2025**, *11*, eadz2218.

[4]	P. Hohenberg, W. Kohn, *Phys. Rev.* **1964**, *136*, B864.

[5]	D. Tian, S. R. Denny, K. Li, H. Wang, S. Kattel, J. G. Chen, *Chem. Soc. Rev.* **2021**, *50*, 12338-12376.

[6]	J. P. Perdew, K. Burke, M. Ernzerhof, *Phys. Rev. Lett*. **1996**, *77*, 3865.

[7]	O. V. Yazyev, I. Tavernelli, L. Helm, U. Röthlisberger, *Phys. Rev. B* **2005**, *71*, 115110.

[8]	S. Kasamatsu, T. Kato, O. Sugino, *Phys. Rev. B* **2017**, *95*, 235120.
